# Contacts in the last 90,000 years over the Strait of Gibraltar evidenced by genetic analysis of wild boar (*Sus scrofa*)

**DOI:** 10.1371/journal.pone.0181929

**Published:** 2017-07-25

**Authors:** Carmen Soria-Boix, Maria P. Donat-Torres, Vicente Urios

**Affiliations:** 1 Estación Biológica Terra Natura, Grupo de Investigación Zoología de Vertebrados, Universidad de Alicante, San Vicente del Raspeig, Alicante, Spain; 2 Instituto de Investigación para la Gestión Integrada de Zonas Costeras, Universidad Politécnica de Valencia, Gandia, Valencia, Spain; University of Florence, ITALY

## Abstract

Contacts across the Strait of Gibraltar in the Pleistocene have been studied in different research papers, which have demonstrated that this apparent barrier has been permeable to human and fauna movements in both directions. Our study, based on the genetic analysis of wild boar (*Sus scrofa)*, suggests that there has been contact between Africa and Europe through the Strait of Gibraltar in the Late Pleistocene (at least in the last 90,000 years), as shown by the partial analysis of mitochondrial DNA. Cytochrome b and the control region from North African wild boar indicate a close relationship with European wild boar, and even some specimens belong to a common haplotype in Europe. The analyses suggest the transformation of the wild boar phylogeography in North Africa by the emergence of a natural communication route in times when sea levels fell due to climatic changes, and possibly through human action, since contacts coincide with both the Last Glacial period and the increasing human dispersion via the strait.

## Introduction

At present, Africa and Europe are in close proximity to each other geographically speaking, and are by only 14 km separated through the Strait of Gibraltar. However, it is known that major falls in sea level (~100 metres) related to glacial periods, and the consequent emergence of islands, reduce this distance to smaller marine barriers, less than 5 km each [1–3]. In this situation the interaction between both sides of the strait seems possible despite it being a barrier for some species [4–8].

Evidence of contacts across major marine distances is not new. Human dispersal across a marine barrier 0.88 million years ago (MYA) has been proven in the Flores Island (Java) [9,10]. During glacial periods, the Strait of Sicily would not have acted as a major geographical barrier for some species [11,12]. On the Strait of Gibraltar, some documented cases are found of movements of hominids and fauna across this permeable barrier [2,10,13–17]. For example, the arrival of humans and vertebrate fauna to the Iberian Peninsula from Africa has been recorded at the sites of Orce (southeast Spain) as early as the Plio-Pleistocene boundary [2,10,18].

Due to the complex biogeographic histories of some species, it may be complicated, or even impossible, to distinguish the cause of movements in the late Pleistocene. The sea level was lower until the Last Glacial Maximum (LGM), some 25,000 and 18,000 years ago. Thus contacts could have taken place by natural migrations or colonisations from anthropogenic introductions [17,19].

In any case, the North African wild boar (*Sus scrofa*) is closely related to the European wild boar, which indicates a strong gene flow [20,21], but no studies exist have focused on the possible routes of these contacts. Very few studies exist about African populations, and the history of the native wild boar in North Africa is poorly known. We found some references from historical and paleontological records about its possible origin [22–24], one study about the genetic structure of the wild boar population of Tunisia [25], and a number of studies about African pigs [26,27]. In GenBank we only found information on three sequences of cytochrome b and four of the control region identified exclusively in Morocco, or which belonged to the sequences also found for wild boars from other areas. In this study, five Moroccan wild boar samples were analysed and incorporated into GenBank [28]. This dataset has sufficed to allow us to study the hypothesis of the present work.

The present study aims to elucidate contacts between Africa and the Iberian Peninsula across the Strait of Gibraltar by considering the genetic similarity of the wild boar populations on both sides of the strait.

We decided to analyse mitochondrial DNA (mtDNA) cytochrome b and the control region because the latter is more hypervariable. The first analysis of Y-chromosome polymorphisms of a Moroccan wild boar is also provided.

## Material and methods

### Samples and DNA extraction

Hair and tissue were obtained from five wild boar individuals. Specifically, four females and one male (WBMoroc2) were sampled from the Middle Atlas in Morocco. Samples were collected during the 2014 and 2015 hunting seasons. Throughout the study area no special permits were required to legally hunt wild boars, only a general hunting licence. Animals were killed for other purposes and no authors were involved in hunting. Samples were obtained directly from licenced hunters.

Mitochondrial DNA was extracted from hair roots and tissue samples using the material and protocols for DNA isolation of the Invisorb® Spin Forensic Kit (STRATEC Biomedical AG, Berlin).

### Mitochondrial DNA amplification and sequencing

Mitochondrial DNA was amplified using the primers and amplification profiles described by Alves et al. [29] (S1 Table). The thermocycling profile was the same for both cytochrome b and the control region: one cycle at 94°C for 2 min, followed by 30 cycles of 94°C for 45 seconds, 55°C for 45 seconds, 72°C for 1 min, and finally an extension step at 72 ºC for 10 min. PCR products were purified and sent to Macrogen (http://www.macrogen.com/eng/) for sequencing. We obtained two complementary fragments for each region, which were assembled using BioEdit 7.2.5 [30].

### Y-chromosome amplification and sequencing

We analysed partial sequences of four Y-chromosome regions using the primers and amplification profiles described by Ramírez et al. [21]. These were carried out under the following conditions: 95°C for 10 min and 35 cycles of 94°C for 1 min, annealing temperature (Tm) (S1 Table) for 1 min, 72°C for 1 min, and finally an extension step at 72°C for 15 min [21,27]. PCR products were purified and sent to Macrogen for sequencing.

### Mitochondrial DNA analyses

In all, 1,152 base pairs (bp) including the entire cytochrome b, were obtained for the five analysed wild boar samples (GenBank accession numbers: KU664546, KU6645407, KU66454, KU664553 and KU608293) and were aligned with the 358 wild boar sequences available in GenBank (Table A in S2 Table). Bearded pig (*Sus barbatus*), celebes wild boar (*Sus celebensis*), philippine warty pig (*Sus philippensis*) and common warthog (*Phacochoerus africanus*) were employed as the outgroups. The partial control region gene (995 bp) was amplified for the five analysed wild boar samples (GenBank accession numbers: KU664554—KU664558) and were aligned with the 1,210 sequences from GenBank (Table B in S2 Table). Celebes wild boar (*S*. *celebensis*) and common warthog (*P*. *africanus*) were employed as outgroups.

The sequences used for the analysis comprised wild boar with a wide geographical distribution, including Europe, Africa, Near East and Asia. These regions represent the geographic areas of interest for our study. From the available GenBank sequences, we selected those from wild boar. We ruled out most sequences from clones, feral pigs, mixed and archaeological specimens, as well as the sequences classified as unverified and predicted. The size of the final dataset to be used for the analyses varied after aligning all the selected sequences and removing the positions that contained gaps and missing data (N).

Sequences were aligned using BioEdit 7.2.5 and ClustalW alignment tool included in this software. The number of haplotypes was calculated using the DnaSP 5.10 software [31]. The cytochrome b haplotypes obtained here were given the code “CB”, and those obtained for the control region were coded as “CR”. The best nucleotide substitution models were selected using jModelTest 2.1.7 [32] under the Bayesian Information Criterion (BIC). The Pairwise genetic distances between sequences were calculated by MEGA6 [33] with 1,000 bootstraps replicates and gamma distribution (shape parameter = 0.5).

Time of divergence (*T*) was estimated using the molecular clock equation *T = K/* (2*r*) [34], where *T* = divergence time in years, *K* = genetic distance and *r* = rate of nucleotide substitutions. The genetic distance (*K*) between *P*. *africanus* and genus *Sus* was calculated with the Tamura 3-parameter model and gamma distribution (shape parameter = 0.5) using MEGA6 in both cytochrome b and the control region. We assumed a substitution rate (r) of 1 x 10−^8^ per site per year for cytochrome b. This rate was previously estimated for complete mtDNA [35]. We used a higher substitution rate (*r*) of 1.37 x 10^−8^ per site per year, as estimated by Pesole et al. [36], for the control region in mammals [37,38].

Bayesian phylogenetic trees were constructed using BEAST 1.8.2 [39]. We assumed a strict clock and the coalescent prior with a constant size. Evolutionary parameters were given by jModeltest. At least two independent Markov chain Monte Carlo (MCMC) chains were run for 50 million generations, and parameter values were sampled every 1,000 generations. We examined the results using Tracer 1.6 [40]. We used TreeAnnotator 1.8.2 [39] to obtain the consensus trees. The first 10% of the sampling trees were ruled out as burn-in and the resulting trees were visualized in FigTree 1.4.2 (http://tree.bio.ed.ac.uk/software/figtree/). Two median-joining networks [41] were generated and visualized using Network 5.0.0.1 (http://www.fluxus-engineering.com). For the cythocrome b, with more complex data than the control region in the final dataset, we used the star contraction option and epsilon = 20.

### Y-chromosome analyses

We obtained a partial sequence of 543 bp of the amelogenin (AMELY) gene, 421 bp intron 24 of the ubiquitin-specific protease 9 (USP9Y) gene, and two fragments that corresponded the ubiquitously transcribed tetratricopeptide repeat gene (UTY). These two fragments belong to 322 bp intron 1 (UTYin1) and 353 bp intron 9 (UTYin9) (GenBank accession numbers: KU664549—KU664552). These were aligned with 11 wild boar sequences from GenBank (Table C in S2 Table) using BioEdit 7.2.5 and ClustalW.

## Results

For methodological reasons, the analyses were carried out with wild boar samples from Europe, Africa, Near East and Asia, but we focused on the analysis of clades that was more related to Africa and Europe. Asian sequences were included to improve the analysis of the divergence times and to better understand the spread of wild boar from Asia to Europe.

The analyses revealed the main mtDNA clades: European (E1), Italian (E2), Near Eastern (NE), and Asian clades (A) [42–44]. In Larson et al. [45], E1 corresponds to clade D1, E2 to D4.

### Cytochrome b analyses

Except for the network (1,030 bp), we used a 897 bp fragment for the analyses that corresponded to the cytochrome b gene. In all, 107 haplotypes were identified from 363 wild boars (S7 Table). The Moroccan and Tunisian samples are included in haplotypes CB9 and CB90 (Table A in S2 Table). CB9 is the commonest haplotype in Europe and is shared by some North African and 33 European (including Italian), four Asian and four wild boars from Near Eastern countries. When focusing on the Iberian Peninsula, we found that on the other side of the Strait of Gibraltar of the 13 Spanish wild boars, 8 were included in this haplotype.

**Fig 1:**
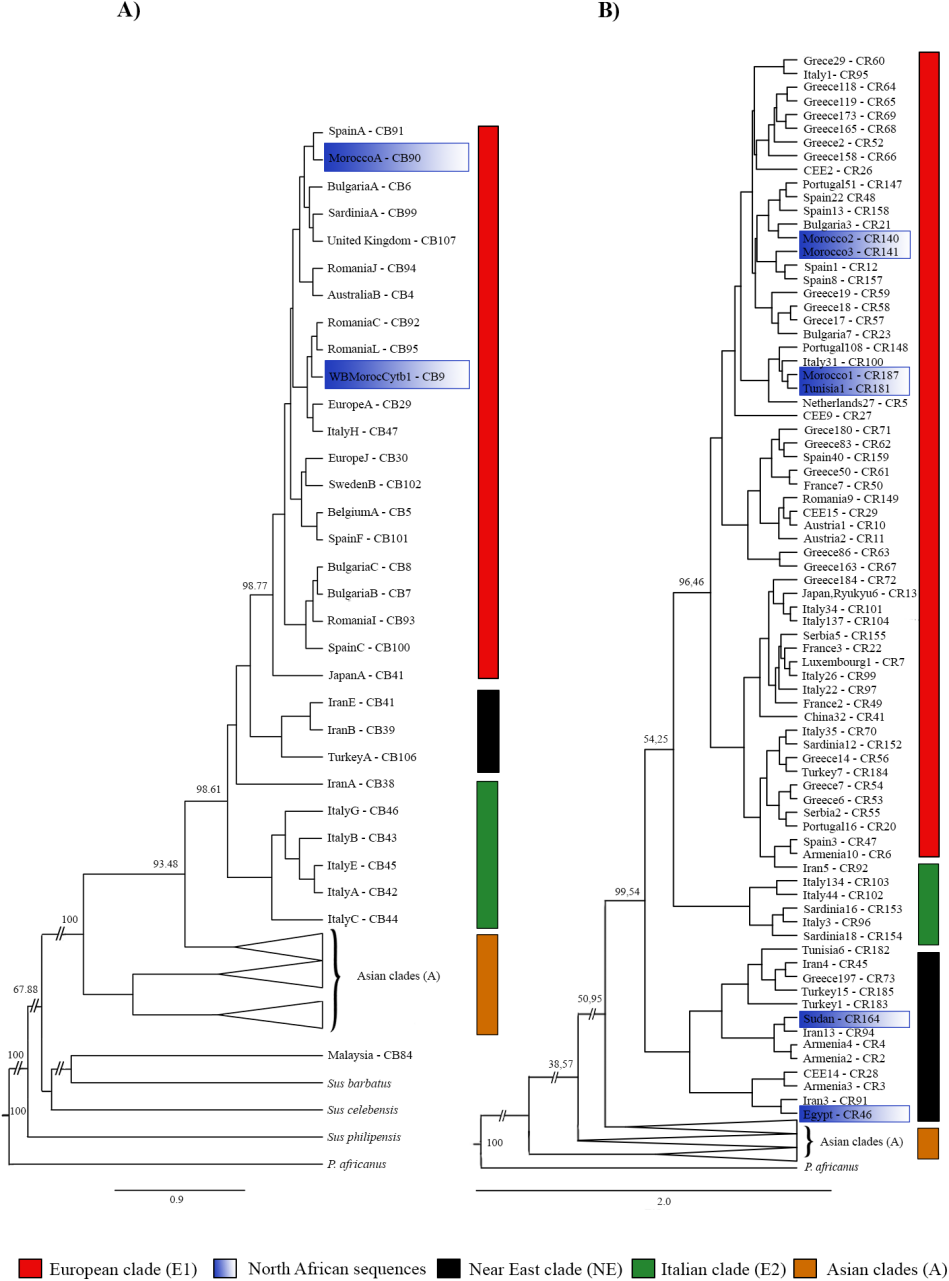
Bayesian phylogenetic trees based on mtDNA. (A) The cytochrome b phylogenetic tree was constructed with 111 haplotypes (897 bp), which represent 363 wild boars and four outgroups. (B) The control region phylogenetic tree was constructed with 188 haplotypes (415 bp), which represented 1,215 wild boars and two outgroups. CEE is the abbreviation for Central and Eastern Europe. In both cases, the numbers above branches indicate posterior values as a percentage (%). Names of sequences correspond to those of the sequences used to represent each haplotype. The haplotype code assigned in this work has been added.

For the phylogenetic analysis, the best model was the Generalised Time-Reversible Evolutionary Model with gamma distribution (GTR + G) [46] for cytochrome b. The Bayesian phylogenetic tree revealed the previously observed clades: E1 (European clade), E2 (Italian clade), NE (Near Eastern clade), A (Asian clade) (Fig 1A). The haplotypes with sequences from Morocco and Tunisia belonged to the European clade (E1).

The pairwise genetic distances between clades ranged from 0.0085 to 0.0138 (Table A in S3 Table). The mean distances between the haplotypes included in the European clade (E1) were calculated (Table B in S3 Table). The MoroccoA haplotype (CB90), exclusive of Africa, differs from the rest by more than CB9, which is the other haplotype with African sequences.

The cytochrome b gene contains six single nucleotide polymorphisms (SNPs) that allow the differentiation between European and Asian haplogroups [26,37,47]. The variable sites are located at positions 873/ 874/ 876/ 879/ 882 and 883 of the sequences from this study, which correspond to positions 15,035/ 15,036/ 15,038/ 15,041/ 15,044 and 15,045 of the pig mtDNA used as a reference (GenBank accession number AJ002189). The Moroccan and Tunisian cytochrome b sequences have European origin (GTGCAG).

In order to understand the genetic diversity in African wild boar populations, variable sites for their sequences were analysed (S5 Table). The cytochrome b sequences (1,047 bp) have seven nucleotide polymorphic sites in the analysed fragment. There are five transitions, one transversion and one deletion.

The time of divergence estimated between genus *Sus* and *P*. *africanus* was 7 million years (between 5.95 and 8.05) for cytochrome b (S6 Table). From the Bayesian phylogenetic tree, it was deduced that the time of isolation between the Asian and the other clades occurred approximately 818,300 years ago. The isolation of the European clade (E1) occurred some 429,500 years ago. The beginning of the isolation of haplotypes found in North Africa took place 63,800 years ago.

**Fig 2:**
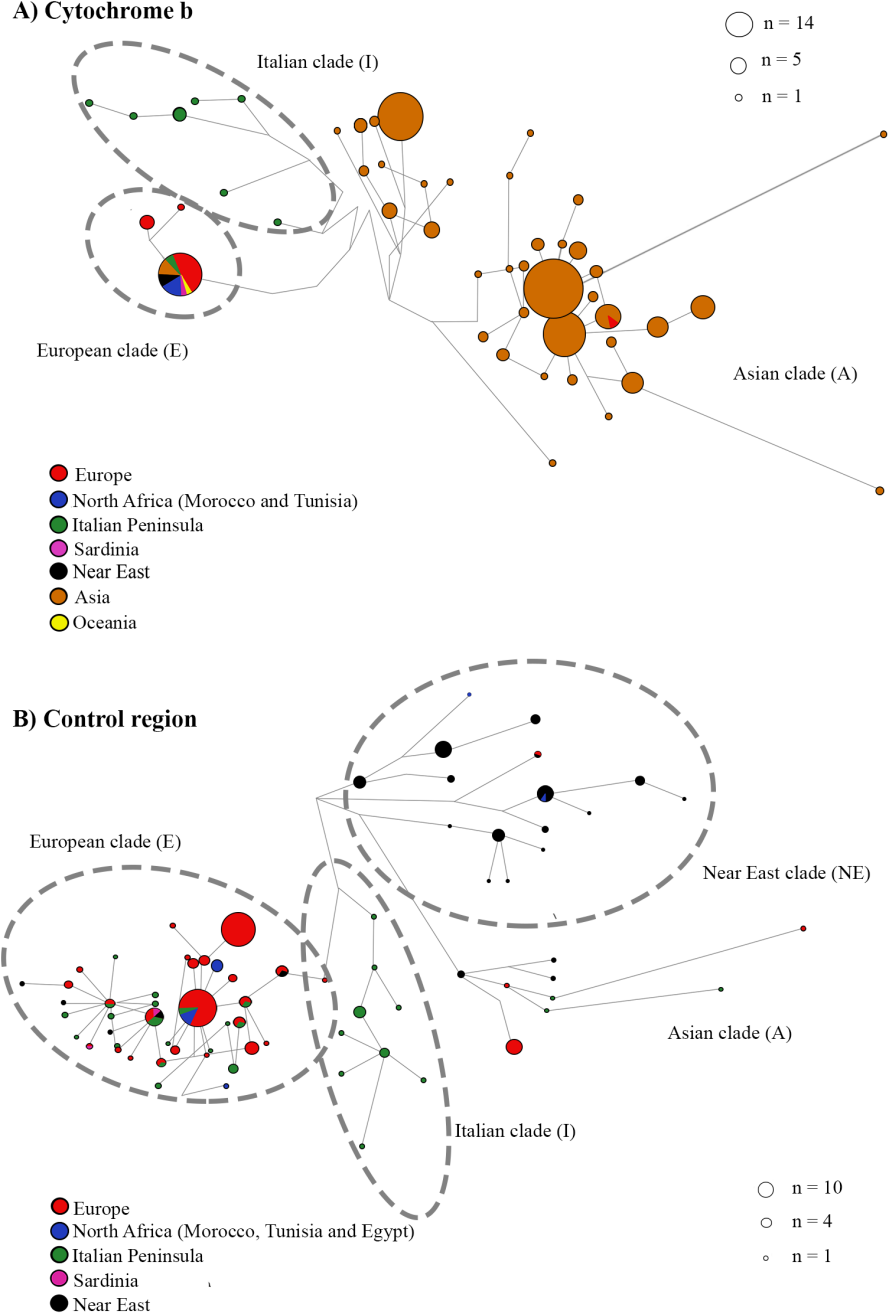
Median-joining networks based on mtDNA. (A) The cytochrome b network was constructed with 308 wild boar sequences (1,030 bp). (B) The control region network was constructed with 328 wild boar sequences (534 bp).

Networks were constructed to better visualize the relationships among clades (Fig 2A). We used fewer sequences with more base pairs to better understand existing relationships and to check for possible variations when using a larger segment size (1,030 bp). North African, Near Eastern and European wild boars clustered in the same way in both the network and the Bayesian phylogenetic tree.

### Control region analyses

We used a 415 bp fragment for the control region analyses, except for the network (534 bp). We obtained 187 haplotypes from 1,215 wild boar sequences (S7 Table) after aligning our sequences with others available in GenBank. Moroccan wild boar belongs to haplotypes CR1, CR140 and CR141 (Table B in S2 Table). CR1 is the commonest haplotype in Europe and is shared by some Moroccan and 274 European (including Italian), three Asian, one Australian (Oceania) and five wild boars from Near Eastern countries. In the Iberian Peninsula, 53 of the 121 wild boars from Portugal, and 24 of the 41 wild boars from Spain, are included in this haplotype. CR1 corresponds to the so-called CB9 in mtDNA cytochrome b. The Tunisian wild boar belongs to haplotypes CR181 and CR182, Egyptian to CR46 and the Sudanese wild boar to CR164.

For the Bayesian phylogenetic analysis, the best model was the Generalised Time-Reversible Evolutionary Model [46], with invariant sites and gamma distribution (GTR + I + G) for the control region. The Bayesian phylogenetic tree revealed the same clades seen in cytochrome b (Fig 1B). The sequences from Morocco and Tunisia clustered in the European clade (E1), except for the CR182 haplotype from Tunisia. The control region sequences from Egypt (CR46), Sudan (CR164) and Tunisia (CR182) belong to the Near Eastern clade (NE). Egypt shares a haplotype with some sequences from Iran.

The pairwise genetic distances between clades ranged from 0.0168 to 0.0293 (Table A in S4 Table). The mean distances between the haplotypes included in the European clade (E1) (Table B in S4 Table) showed that there was a longer distance between the haplotypes found in Morocco (between CR1 and CR 140/141) than between some Moroccan ones and the Tunisian one (between CR1 and CR181). The higher values in the mtDNA control region distances can be explained by it being a more hypervariable region than cytochrome b.

The analysed partial control region (406 bp) has 12 nucleotide polymorphic sites, including 11 transitions and one deletion (S5 Table).

The time of divergence estimated between genus *Sus* and *P*. *africanus* was 6.75 million years (between 5.51 and 7.99) for the control region. The time of the isolation between the Asian and the other clades occurred approximately 1,234,100 years ago (S6 Table). The time of divergence of the European clade (E1) was 696,000 years ago, and the beginning of the isolation of the haplotypes found in North Africa took place 116,600 years ago, according to the phylogenetic tree.

Finally, we generated the network (Fig 2B). In this case we included only the sequences that belonged to clades E1 (Europe), E2 (Italy) and NE (Near East) to focus on the connections of these groups. The relationships that we found were similar for both the network and the Bayesian phylogenetic tree.

### Y-chromosome haplotype

For the Y-chromosome analysis, we sequenced the partial AMELY, USP9Y and UTY (UTYin1 and UTYin9) regions in one male from Morocco (WBMoroc2) in order to compare them with the published sequences of previous studies [21,48,49] and to identify their haplotype. There were three defined haplotypes, and our results showed that, according to Ramírez et al. [21], our sample belonged to the HY2 haplotype.

## Discussion

We explored the relationships via the Strait of Gibraltar based on an analysis of the partial mtDNA of wild boar (*S*. *scrofa*). We agree with the presence of one European clade (E1) that is widely distributed in Europe and North Africa (Morocco and Tunisia), one exclusive of Italy (E2), and another with most Near Eastern sequences (NE). These results are congruent with those reported by Larson et al. [45] and Meiri et al. [50].

The control region phylogenetic tree also shows some Asian haplotypes in the basal clade. This coincides with the results of Larson et al. [45]. Regarding genetic distances, in both cases the European clade is closer to the clade of the Near East than to the Italian one. When considering the distances only in cytochrome b, the Asian clade displays the same distance with both the Italian and the Near East one, which gives rise to the different distribution in the corresponding trees. However, the cytochrome b shows some Asian sequences that are closer to the European ones, which is due to the specific dispersal process of these populations and their contacts with others [51].

Finally, the analyses confirm that the modern wild boars from Morocco and Tunisia share European haplotypes. Only the Tunisian wild boar with haplotype CR182, which is less present in our results, belongs to the Near Eastern clade as wild boars from Egypt and Sudan. The median-joining networks showed the same relationships when fewer sequences with more base pairs were used.

We obtained an interesting finding for the Moroccan wild boar (Table B in S4 Table). The estimated genetic distance between the sequences belong to the CR1 haplotype from Morocco and the Tunisian CR181 is 0.0025. The estimated distance with the CR140 and CR141 haplotypes from Morocco is 0.0051 and 0.0077 respectively. CR1 also display shorter distances (0.0025) with some haplotypes found in Portugal, Spain, and even Greece (haplotypes CR12, 21, 48, 52, 60, 100, 148). A long distance is seen between haplotypes CR141 and CR142. Differences between Moroccan populations are consistent with results from previous studies, and might indicate different origins or isolation due to geographical barriers in Maghreb [6,52]. However, very little information is available in other studies on samples used from Morocco to obtain mtDNA. The specimens that belong to our study (CR1) are from the province of Khenifra. Morocco1 (CR1) is from Taforalt (Oujda) [45] and Morocco2 (CR140) and Morocco4 (CR1) are labelled with the location Atlas/Rabat [53]. Although these last two samples are referenced with the same location, this covers a wide area and could belong to different populations. As we do not have further information, and without knowing the location of Morocco3 (CR142), more samples will be needed to confirm the hypothesis.

When focusing on the sequences from Israel, we find that they are included in the European clade (E1) and within the most frequent haplotype (CB9). Larson et al. [45] obtained a similar result for one wild boar from Armenia, and suggested that it might be due to introgression from European wild boars or feral pigs. This latter possibility does not seem plausible because Far Eastern haplotypes have a 29% frequency in International pig breeds [8,37], but there is no evidence for these haplotypes appearing in wild boars from Armenia, Israel or North Africa. The most logical explanation is the interchange of haplotypes between wild boars through human action or natural movements. In any case, even when the near Eastern and African wild boars share this haplotype, it does not seem directly related to one other. Neither the phylogenetic trees nor networks indicate any similarity between both populations, except for one haplotype from Tunisia (CR182), which is located in the Near Eastern clade. Therefore, the commonest haplotype might have been transmitted to North Africa by contacts with the European wild boar.

The six SNPs located at specific mtDNA cytochrome b positions, and used to differentiate the origin of samples, confirm that the origin of all the sequences from Morocco and Tunisia used for these analyses is European. The results of the variable sites in the African wild boar sequences are representative of a strong transitional bias, usually found in mammals’ mitochondrial evolution [54–56].

From the genetic distances and Bayesian phylogenetic trees included in this study, we can estimate that the Near Eastern clade originated between 857,100 and 429,500 years ago (S6 Table). After this event, the wild boar arrived in North Africa possibly through Egypt, which would have isolated it from the Near Eastern clade. Presence of wild boar fossils from the Middle Pleistocene in North Africa and in several Near Eastern countries [24,57] supports our results, and the idea of wild boar forming part of fauna of Maghreb during this period. Nevertheless, the North African wild boar currently has European haplotypes.

Since the control region is more hypervariable than cytochrome b to differences like the size of the mtDNA segment used, and the fact that the analysed specimens of both regions do not belong to the same specimens in most cases, the years of divergence shown in S5 Table vary within a narrow range. For the haplotypes found in Morocco and Tunisia, our analyses gave an approximation of their isolation of 63,800 years ago for cytochrome b, and of 116,600 years ago for the control region. Not until these haplotypes appeared could their dispersion between Europe and Africa have begun, which caused the genetic similarity. Therefore, by taking into account all these conditions and the years obtained, we offer an average approximation of 90,000 years (in the Late Pleistocene) for the time when a major genetic flow started between populations on both sides of the Strait of Gibraltar. This process must have given rise to the modern African wild boar, and something seems to have happened in Israel [50].

The Y-chromosome analysis result shows that our sample belongs to haplotype HY2, and is present in at least Tunisia, Spain, Russia, Iran and Japan. Haplotypes HY1 and HY2 have been documented in Tunisia [21]. With the information available from the Moroccan and Tunisian Y-chromosome sequences, it seems that they might be related to the European or Near Eastern wild boar. Haplotype HY3 is relatively abundant in Far Eastern specimens, and has been detected in Kenyan and Mukota pigs [21], but is absent in the North African wild boar. These results support the hypothesis that there was no direct genetic flow between Asian and African wild boars, which would be congruent with the fact that the commonest haplotype in mtDNA would not be in Africa due to introgression by pigs, as previously mentioned. Although these data are not enough to draw definitive conclusions, given the lack of patrilinear history information on this point in Morocco, we feel the analysis has been valuable.

### The role of the Strait of Gibraltar as a marine permeable barrier between Europe and Africa

The results are interesting because it would be more logical for North African wild boars to share their haplotypes with those of Egypt, Sudan, or even with those of the Near East, due to connectivity by land. However, all their haplotypes are European, except for CR182.

According to Manlius and Gautier [57], the so-called wild boars from Sudan are feral pigs, and the modern wild boars from Egypt were probably introduced by humans in the Neolithic period, like sheep and goats. Even so, presence of native wild boar at low densities in the past cannot be ruled out, and natural colonisation through Egypt is logical and supported by the existence of fossils found in North Africa. Accordingly, if the only contacts had been made by land through Near East countries, the African wild boar would form part of the Near Eastern clade, or would at least differ from European populations.

Therefore, isolation between populations from Egypt and North Africa in the past (in the Late Pleistocene) and contacts with the European wild boar across the Strait of Gibraltar, the most likely route, could have had a strong effect on the mtDNA of the African populations. Obviously, we cannot rule out previous contacts to the Late Pleistocene during former glacial periods. The absence of a genetic footprint could be due to the genetic flow not being enough to have endured [49]. The CR182 haplotype found in Tunisia should be the result of either genetic introgression with the Near East wild boar or a trace from the past. Indeed this haplotype is older than the rest by at least 81,000 years.

Contacts across the Strait of Gibraltar could have been possible during glacial periods when sea level fell and it was easier to cross. The Last Glacial Maximum, the maximum extent of glaciation during the Last Glacial period, occurred during the interval between 25,000 and 18,000 years ago [17,19], and the Last Glacial period finished 11,700 years ago. The Late Glacial, the beginning of the modern warm period, began approximately 13,000 years ago [58], but the rise in temperatures was gradual. During cold periods, the currents in the strait would have been minimal or inexistent, which would have facilitated crossing the strait [2].

As for the causes of movements, if they took place approximately from 90,000 onward, it is difficult to know if contacts were made by natural colonisations, by human action, or by a combination of both without having further data [48,49]. This is due to several events occurring at the same time. In addition, it would appear that the South region of the Iberian Peninsula and North Africa form part of a refugial subcentre denominated Atlanto-Mediterranean, from which species could have recolonised areas in North of Europe at the beginning of the post-glacial periods the [5,11,17].

In fact the existence of human contacts across the strait during the period between 12,000 and 10,500 years ago has been proven, which was when sea levels were still rising [13,59]. Since then human contacts have happened. The existence of cattle of one characteristic haplotype of African breeds in the Iberian Bronze Age suggests that contacts over the Strait of Gibraltar were caused by interactions among communities, their culture and livestock in prehistory [14]. Besides, other animals like genet (*Genetta genetta*), barbary ape (*Macaca sylvanus*) or egyptian mongoose (*Herpestes ichneumon*) are accepted as introductions between Europe and North Africa [24]. Movements of people between both continents, who took pigs with them from the 15th century onward on exploratory or commercial routes, and with the consequent genetic introgression, is another possible explanation for similarity [20,21]. In our case it is more likely that migrations occurred naturally. Accessible information suggests that people took domestic varieties of livestock, such as cattle or pigs, but no information on transporting wild boar is available.

Similarly, the arrival of the wild boar from mainland Asia to the Ryukyu islands in the Late Pleistocene seems to be a fact [51]. A much closer geographic proximity between the two regions when sea levels were lower could account for this dispersion.

Finally, a strong gene flow might have been persistent over time, and a population decline or a displacement of the original population, followed by the expansion of new haplotypes, could have occurred. As a result, the African wild boar forms part of the European clade, at least it does according to its mtDNA.

Therefore, we suggest that the Strait of Gibraltar acted as a bridge for wild boar (*S*. *scrofa*) to disperse in the Late Pleistocene. In this case, the dispersion of specimens accompanied by a strong gene flow would have been occurred from at least 90,000 years onward. Genetic analyses, the history of *S*. *scrofa*, and the fact that the Last Glacial period finished 11,700 years ago, all suggest natural dispersion, but we cannot rule out the contact by human action.

## Supporting information

S1 Table Primers used for DNA amplification. (DOCX)

S2 Table Tables with information about the sequences obtained from GenBank and those from this study. Tables with information about cytochrome b (Table A), the control region (Table B) and the Y-chromosome (Table C) sequences. The original code, country of origin, species, state, accession numbers, haplotypes, clades and authors have been indicated.
(XLSX)

S3 Table The cytochrome b pairwise distances between sequences. (A) The pairwise distances between clades are shown. (B) The pairwise distances between the haplotypes that belong to the European clade (E1).
(DOCX)

S4 Table The control region pairwise distances between sequences. (A) The pairwise distances between clades are shown. (B) The pairwise distances between the haplotypes that belong to the European clade (E1).
(DOCX)

S5 Table Variable sites of the sequences obtained from Moroccan and Tunisian wild boars. (DOCX)

S6 Table Estimated time of divergence calculated for each clade with strict clock. (DOCX)

S7 Table Number of detected haplotypes in this study of wild boar (*Sus scrofa*), and its frequency. (A) Cytochrome b haplotypes (B) Control region haplotypes.
(XLSX)

## References

[1] Straus LG. Africa and Iberia in the Pleistocene. Quat Int. 2001 Jan; 75(1):91–102. doi: 10.1016/S1040-6182(00)00081-1

[2] Gibert J, Gibert L, Iglesias A. The Gibraltar Strait: A Pleistocene door of Europe? Hum Evol. 2003; 18(3):147–60. doi: 10.1007/BF02436283

[3] Phoca-Cosmetatou N, Rabett RJ. Pleistocene Island Occupation in the Mediterranean: Insights from a Tied-Biome Approach to Glacial Refugia In Boyle K, Rabett RJ, Hunt CO, editors, Living in the Landscape: Essays in Honour of Graeme Barker. Cambridge: McDonald Institute for Archaeological Research 2014 p. 83–108. (McDonald Institute Monograph Series).

[4] Castella V, Ruedi M, Excoffier L, Ibáñez C, Arlettaz R, Hausser J. Is the Gibraltar Strait a barrier to gene flow for the bat *Myotis myotis* (Chiroptera: Vespertilionidae)? Mol Ecol. 2000; 9(11):1761–72. doi: 10.1046/j.1365-294x.2000.01069.x 11091312 10.1046/j.1365-294x.2000.01069.x

[5] Gantenbein B, Largiadèr CR. The phylogeographic importance of the Strait of Gibraltar as a gene flow barrier in terrestrial arthropods: a case study with the scorpion Buthus occitanus as model organism. Mol Phylogenet Evol. 2003; 28(1):119–30. doi: 10.1016/S1055-7903(03)00031-9 12801475 10.1016/s1055-7903(03)00031-9

[6] Schmitt T. Molecular biogeography of Europe: Pleistocene cycles and postglacial trends. Front Zool. 2007; 4(1):11 PMC1868914 doi: 10.1186/1742-9994-4-11 17439649 10.1186/1742-9994-4-11 PMC1868914

[7] Jl García-Mudarra, Ibáñez C, Juste J. The Straits of Gibraltar: barrier or bridge to Ibero-Moroccan bat diversity? Biol J Linn Soc. 2009; 96(2):434–50. doi: 10.1111/j.1095-8312.2008.01128.x

[8] Horreo JL, Alonso JC, Palacín C, Milá B. Genetic structure in Iberian and Moroccan populations of the globally threatened great bustard Otis tarda: a microsatellite perspective. J Avian Biol. 2014; 45(5):507–13. doi: 10.1111/jav.00401

[9] Brumm A, Jensen GM, van den Bergh GD, Morwood MJ, Kurniawan I, Aziz F, et al Hominins on Flores, Indonesia, by one million years ago. Nature. 2010 4 1; 464(7289):748–52. doi: 10.1038/nature08844 20237472 10.1038/nature08844

[10] Gibert L, Scott GR, Scholz D, Budsky A, Ferràndez C, Ribot F, et al Chronology for the Cueva Victoria fossil site (SE Spain): Evidence for Early Pleistocene Afro-Iberian dispersals. J Hum Evol. 2016; 90: 183–97. doi: 10.1016/j.jhevol.2015.08.002 26581114 10.1016/j.jhevol.2015.08.002

[11] Habel JC, Dieker P, Schmitt T. Biogeographical connections between the Maghreb and the Mediterranean peninsulas of southern Europe. Biol J Linn Soc. 2009; 98: 693–703

[12] Habel JC, Rödder D, Stefano S, Meyer M, Schmitt T. Strong genetic cohesiveness between Italy and North Africa in four butterfly species. Biol J Linn Soc. 2010; 99(4):818–30. doi: 10.1111/j.1095-8312.2010.01394.x

[13] Rhouda T, Martínez-Redondo D, Gómez-Durán A, Elmtili N, Idaomar M, Díez-Sánchez C, et al Moroccan mitochondrial genetic background suggests prehistoric human migrations across the Gibraltar Strait. Mitochondrion. 2009; 9(6):402–7. doi: 10.1016/j.mito.2009.07.003 19631765 10.1016/j.mito.2009.07.003

[14] Anderung C, Bouwman A, Persson P, Carretero JM, Ortega AI, Elburg R, et al Prehistoric contacts over the Straits of Gibraltar indicated by genetic analysis of Iberian Bronze Age cattle. Proc Natl Acad Sci United States Am. 2005 6 14; 102(24): 8431–5. PMC1150856 doi: 10.1073/pnas.0503396102 15941827 10.1073/pnas.0503396102 PMC1150856

[15] Ramos-Muñoz J, Cantillo-Duarte JJ, Bernal-Casasola D, Barrena-Tocino A, Domínguez-Bella S, Vijande-Vila E, et al Early use of marine resources by Middle/Upper Pleistocene human societies: The case of Benzú rockshelter (northern Africa). Quat Int. 2016; 407, Part B:6–15. doi: 10.1016/j.quaint.2015.12.092

[16] Graciá E, Giménez A, Anadón JD, Harris DJ, Fritz U, Botella F. The uncertainty of Late Pleistocene range expansions in the western Mediterranean: a case study of the colonization of south-eastern Spain by the spur-thighed tortoise, Testudo graeca. J Biogeogr. 2013; 40(2):323–34. doi: 10.1111/jbi.12012

[17] Husemann M, Schmitt T, Zachos FE, Ulrich W, Habel JC. Palaearctic biogeography revisited: evidence for the existence of a North African refugium for Western Palaearctic biota. J Biogeogr. 2014; 41(1):81–94. doi: 10.1111/jbi.12180

[18] Gibert J, Gibert L, Iglesias A, Maestro E. Two “Oldowan” assemblages in the Plio-Pleistocene deposits of the Orce region, southeast Spain. Antiquity. 1998; 72(275):17–25. doi: 10.1017/S0003598X00086233

[19] Mikolajewicz U. Modeling Mediterranean Ocean climate of the Last Glacial Maximum. Clim Past. 2011; 7: 161–180. doi: 10.5194/cp-7-161-2011

[20] Ojeda A, Rozas J, Folch JM, Pérez-Enciso M. Unexpected High Polymorphism at the FABP4 Gene Unveils a Complex History for Pig Populations. Genetics. 2006 12 7; 174(4): 2119–27. PMC1698616 doi: 10.1534/genetics.106.063057 17057239 10.1534/genetics.106.063057 PMC1698616

[21] Ramírez O, Ojeda A, Tomàs A, Gallardo D, Huang LS, Folch JM, et al Integrating Y-chromosome, mitochondrial, and autosomal data to analyze the origin of pig breeds. Mol Biol Evol. 2009; 26(9): 2061–72. doi: 10.1093/molbev/msp118 19535739 10.1093/molbev/msp118

[22] Gharaibeh BM. Systematics, distribution, and zoogeography of mammals of Tunisia [dissertation]. Graduate Faculty of Texas Tech University; 1997. Available from: https://ttu-ir.tdl.org/ttu-ir/handle/2346/9796

[23] Dobson M. Mammal distributions in the western Mediterranean: the role of human intervention. Mamm Rev. 1998 6 1;28(2): 77–88. doi: 10.1046/j.1365-2907.1998.00027.x

[24] Blench RM. A history of pigs in Africa In: Blench RM, Mac Donald K, editors. Origins and development of African livestock: archaeology, genetics, linguistics and ethnography. Florence, KY: Routledge Books; 1999 p. 355–367

[25] Hajji GEM, Zachos FE. Mitochondrial and nuclear DNA analyses reveal pronounced genetic structuring in Tunisian wild boar *Sus scrofa*. Eur J Wildl Res. 2011; 57(3): 449–56. doi: 10.1007/s10344-010-0452-3

[26] Olalde I, Capote J, Del-Arco MC, Atoche P, Delgado T, González-Anton R, et al Ancient DNA sheds light on the ancestry of pre-hispanic Canarian pigs. Genet Sel Evol. 2015; 47(1): 1–5. PMC4421913 doi: 10.1186/s12711-015-0115-7 25944642 10.1186/s12711-015-0115-7 PMC4421913

[27] Noce A, Amills M, Manunza A, Muwanika V, Muhangi D, Aliro T, et al East African pigs have a complex Indian, Far Eastern and Western ancestry. Anim Genet. 2015 8 1; 46(4): 433–6. doi: 10.1111/age.12305 26011180 10.1111/age.12305

[28] Benson DA, Karsch-Mizrachi I, Clark K, Lipman DJ, Ostell J, Sayers EW. GenBank. Nucleic Acids Res. Oxford University Press; 2012 1 5; 40 (Database issue):D48–53. PMC3245039 doi: 10.1093/nar/gkr1202 22144687 10.1093/nar/gkr1202 PMC3245039

[29] Alves E, Ovilo C, Rodriguez MC, Silio L. Mitochondrial DNA sequence variation and phylogenetic relationships among Iberian pigs and other domestic and wild pig populations. Anim Genet. 2003 10; 34(5): 319–24. doi: 10.1046/j.1365-2052.2003.01010.x 14510666 10.1046/j.1365-2052.2003.01010.x

[30] Hall TA. BioEdit: a user-friendly biological sequence alignment editor and analysis program for Windows 95/98/NT. Nucl. Acids. Symp. Ser; 1999 41: 95–98

[31] Librado P, Rozas J. DnaSP v5: a software for comprehensive analysis of DNA polymorphism data. Bioinforma. 2009 6 1; 25(11):1451–2. doi: 10.1093/bioinformatics/btp187 19346325 10.1093/bioinformatics/btp187

[32] Darriba D, Taboada GL, Doallo R, Posada D. jModelTest 2: more models, new heuristics and high-performance computing. Nat Methods. 2012 8 30; 9(8): 772 PMC4594756 doi: 10.1038/nmeth.2109 22847109 10.1038/nmeth.2109 PMC4594756

[33] Tamura K, Stecher G, Peterson D, Filipski A, Kumar S. MEGA6: Molecular Evolutionary Genetics Analysis version 6.0. Mol Biol Evol. 2013 10 16; 30(12): 2725–2729. PMC3840312 doi: 10.1093/molbev/mst197 24132122 10.1093/molbev/mst197 PMC3840312

[34] Li WH. Molecular evolution 1st ed. Massachusetts: Sinauer Associates; 1997

[35] Brown WM, George M, Wilson AC. Rapid evolution of animal mitochondrial DNA. Proc Natl Acad Sci. 1979 4 1; 76(4): 1967–71. ; PMC383514 109836 10.1073/pnas.76.4.1967 PMC383514

[36] Pesole G, Gissi C, De Chirico A, Saccone, C. Nucleotide substitution rate of mammalian mitochondrial genomes. J. Mol. Evol. 1999; 48(4): 427–434. doi: 10.1007/Pl00006487 10079281 10.1007/pl00006487

[37] Fang M, Andersson L. Mitochondrial diversity in European and Chinese pigs is consistent with population expansions that occurred prior to domestication. Proc R Soc B Biol Sci. 2006 7 22; 273(1595): 1803–10. PMC1634785 doi: 10.1098/rspb.2006.3514 16790414 10.1098/rspb.2006.3514 PMC1634785

[38] Fernández AI, Alves E, Óvilo C, Rodríguez MC, Silió L. Divergence time estimates of East Asian and European pigs based on multiple near complete mitochondrial DNA sequences. Anim Genet. 2011 2 1; 42(1): 86–8. doi: 10.1111/j.1365-2052.2010.02068.x 20477794 10.1111/j.1365-2052.2010.02068.x

[39] Drummond AJ, Suchard MA, Xie D, Rambaut A. Bayesian Phylogenetics with BEAUti and the BEAST 1.7. Mol Biol Evol. 2012 8 25; 29(8): 1969–73. PMC3408070 doi: 10.1093/molbev/mss075 22367748 10.1093/molbev/mss075 PMC3408070

[40] Rambaut A, Suchard MA, Xie D, Drummond AJ. Tracer v1.6. 2014. Available from http://beast.bio.ed.ac.uk/Tracer

[41] Bandelt HJ, Forster P, Röhl A. Median-joining networks for inferring intraspecific phylogenies. Mol Biol Evol. 1999 1 1; 16(1): 37–48. doi: 10.1093/oxfordjournals.molbev.a026036 10331250 10.1093/oxfordjournals.molbev.a026036

[42] Scandura M, Iacolina L, Crestanello B, Pecchioli E, Di Benedetto MF, Russo V, et al Ancient vs. recent processes as factors shaping the genetic variation of the European wild boar: Are the effects of the last glaciation still detectable? Mol Ecol. 2008; 17(7): 1745–62. doi: 10.1111/j.1365-294X.2008.03703.x 18371016 10.1111/j.1365-294X.2008.03703.x

[43] Giuffra E, Kijas JM, Amarger V, Carlborg O, Jeon JT, Andersson L. The origin of the domestic pig: independent domestication and subsequent introgression. Genetics. 2000 4; 154(4): 1785–91. ; PMC1461048 10747069 10.1093/genetics/154.4.1785 PMC1461048

[44] van Asch B, Pereira F, Santos LS, Carneiro J, Santos N, Amorim A. Mitochondrial lineages reveal intense gene flow between Iberian wild boars and South Iberian pig breeds. Anim Genet. 2012; 43(1): 35–41. doi: 10.1111/j.1365-2052.2011.02222.x 22221023 10.1111/j.1365-2052.2011.02222.x

[45] Larson G, Dobney K, Albarella U, Fang M, Matisoo-Smith E, Robins J, et al Worldwide Phylogeography of Wild Boar Reveals Multiple Centers of Pig Domestication. Science (80-). 2005 3 10; 307(5715): 1618 LP–1621. doi: 10.1126/science.1106927 15761152 10.1126/science.1106927

[46] Tavaré S. Some Probabilistic and Statistical Problems in the Analysis of DNA Sequences In American Mathematical Society: Lectures on Mathematics in the Life Sciences. AMS. 1986; 17:57–86.

[47] Clop A, Amills M, Noguera JL, Fernández A, Capote J, Ramón MM, et al Estimating the frequency of Asian cytochrome B haplotypes in standard European and local Spanish pig breeds. Genet Sel Evol. 2004 1 15; 36(1): 97–104. PMC2697182 doi: 10.1186/1297-9686-36-1-97 14713412 10.1186/1297-9686-36-1-97 PMC2697182

[48] Iwase M, Satta Y, Hirai Y, Hirai H, Imai H, Takahata N. The amelogenin loci span an ancient pseudoautosomal boundary in diverse mammalian species. Proc Natl Acad Sci U S A. 2003 4 29; 100(9): 5258–63. PMC154332 doi: 10.1073/pnas.0635848100 12672962 10.1073/pnas.0635848100 PMC154332

[49] Cliffe KM, Day AE, Bagga M, Siggens K, Quilter CR, Lowden S, et al Analysis of the non-recombining Y chromosome defines polymorphisms in domestic pig breeds: ancestral bases identified by comparative sequencing. Anim Genet. 2010 12 1; 41(6): 619–29. doi: 10.1111/j.1365-2052.2010.02070.x 20477804 10.1111/j.1365-2052.2010.02070.x

[50] Meiri M, Huchon D, Bar-Oz G, Boaretto E, Horwitz LK, Maeir AM, et al Ancient DNA and Population Turnover in Southern Levantine Pigs- Signature of the Sea Peoples Migration? Sci Rep. 2013 11 4; 3: 3035 PMC3816294 doi: 10.1038/srep03035 24186332 10.1038/srep03035 PMC3816294

[51] Yoshikawa S, Mimura M, Watanabe S, Lin LK, Ota H, Mizoguchi Y. Historical Relationships among Wild Boar Populations of the Ryukyu Archipelago and Other Eurasian regions, as Inferred from Mitochondrial Cytochrome b Gene Sequences. Zoolog Sci. 2016; 33(5): 520–526. doi: 10.2108/zs160025 27715420 10.2108/zs160025

[52] Juste J, Bilgin R, Munoz J, Ibanez C. Mitochondrial DNA signatures at different spatial scales: from the effects of the Straits of Gibraltar to population structure in the meridional serotine bat *(Eptesicus isabellinus)*. Heredity (Edinb). 2009 4 29; 103(2):178–87. doi: 10.1038/hdy.2009.47 19401715 10.1038/hdy.2009.47

[53] Vilaça ST, Biosa D, Zachos F, Iacolina L, Kirschning J, Alves PC, et al Mitochondrial phylogeography of the European wild boar: the effect of climate on genetic diversity and spatial lineage sorting across Europe. J Biogeogr. 2014 1 27; 41(5): 987–998. doi: 10.1111/jbi.12268

[54] Vigilant L, Stoneking M, Harpending H, Hawkes K, Wilson AC. African populations and the evolution of human mitochondrial DNA. Science (80-). 1991 9 27; 253(5027): 1503 LP–1507. 1840702 10.1126/science.1840702

[55] Kim K-1I, Yang Y-H, Lee S-S, Park C, Ma R, Bouzat JL, et al Phylogenetic relationships of Cheju horses to other horse breeds as determined by mtDNA D-loop sequence polymorphism. Anim Genet. 1999 4 1; 30(2): 102–8. doi: 10.1046/j.1365-2052.1999.00419.x 10376300 10.1046/j.1365-2052.1999.00419.x

[56] Kim K-I, Lee J-H, Li K, Zhang Y-P, Lee S-S, Gongora J, et al Phylogenetic relationships of Asian and European pig breeds determined by mitochondrial DNA D-loop sequence polymorphism. Anim Genet. 2002; 33(1): 19–25. doi: 10.1046/j.1365-2052.2002.00784.x 11849133 10.1046/j.1365-2052.2002.00784.x

[57] Manlius N, Gautier A. The wild boar of Egypt. C R Acad Sci III. 1999 7; 322(7):573–7. 10488431 10.1016/s0764-4469(00)88527-3

[58] Matthiesen S, Haines K. A hydraulic box model study of the Mediterranean response to postglacial sea-level rise. Paleoceanography. 2003; 18(4):1–12. doi: 10.1029/2003PA000880

[59] Hernández CL, Soares P, Dugoujon JM, Novelletto A, Rodríguez JN, Rito T, et al Early Holocenic and Historic mtDNA African Signatures in the Iberian Peninsula: The Andalusian Region as a Paradigm. PLoS One. 2015; 10(10):1–24. PMC4624789 doi: 10.1371/journal.pone.0139784 26509580 10.1371/journal.pone.0139784 PMC4624789

