# Supplementary material for: Contacts in the last 90,000 years over the Strait of Gibraltar evidenced by genetic analysis of wild boar (Sus scrofa)
Source: PLoS One. 2017 Jul 25;12(7):e0181929. doi: 10.1371/journal.pone.0181929 (PMC5526546; doi:10.1371/journal.pone.0181929)
Supplement: S1 Table — (DOCX) [file pone.0181929.s001.docx]

**S1 Table. Primers used for DNA amplification**

Sequence: All primers are listed 5’ to 3’

Tm (ºC): Annealing temperature

Ref. Article: Previous studies consulted for the preparation of primers and PCRs

| **CONTROL REGION** | | |  |  |  |  |
| --- | --- | --- | --- | --- | --- | --- |
|  |  | |  |  |  |  |
| Oligo Name |  | | Sequence | Tm (ºC) | Ref. Article | Size |
| CR511 | Forward | | CGCCATCAGCACCCAAAGCT | 55 | Alves et al. 2003 | 511 |
|  | Reverse | | TGGGCGATTTTAGGTGAGATGGT |  |  |  |
|  |  | |  |  |  |  |
| CR810 | Forward | | CCGTGGGGGTTTCTATTGA | 55 | Alves et al. 2003 | 810 |
|  | Reverse | | ATTTTGGGAGGTTATTGTGTTGTA |  |  |  |
|  |  | |  |  |  |  |
|  | | |  |  |  |  |
| **CYTOCHROME B** | | |  |  |  |  |
|  |  | |  |  |  |  |
| Oligo Name |  | | Sequence | Tm (ºC) | Ref. Article | Size |
| CytB1 | Forward | | CACGACCAATGACATGAAAAATC | 55 | Alves et al. 2003 | 635 |
|  | Reverse | | GCTGCGAGGGCGGTAAT |  |  |  |
|  |  | |  |  |  |  |
| CytB2 | Forward | | TCTTCGCCTTCCACTTTATCCTG | 55 | Alves et al. 2003 | 661 |
|  | Reverse | | TGGCCCTCCTTTTCTGGTTTA |  |  |  |
|  |  | |  |  |  |  |
|  | | |  |  |  |  |
| **Y-CHROMOSOME** | | |  |  |  |  |
|  | |  |  |  |  |  |
| Oligo Name | |  | Sequence | Tm (ºC) | Ref. Article | Size |
| AMELY | | Forward | GCGTTACATGCATATTGCCTTG | 55 | Ramírez et al. 2009 | 543 |
|  |  | Reverse | TCAAGGATGCTGGAGCTTTT |  |  |  |
|  | |  |  |  |  |  |
| USP9Y in24 | | Forward | GGACTGGTTCTTAATCAGGGTAATTTA | 65 | Ramírez et al. 2009 | 427 |
|  |  | Reverse | TAGCACCACTGCTTGATCATGAG |  |  |  |
|  | |  |  |  |  |  |
| UTYin1 | | Forward | AGCTGTTTTCGGTGATGAGG | 60 | Ramírez et al. 2009 | 330 |
|  |  | Reverse | TGCCCAACAGAGTTTTAGTCC |  |  |  |
|  | |  |  |  |  |  |
| UTYin9 | | Forward | GGCCTTACCCAAGGCATGTAG | 65 | Ramírez et al. 2009 | 353 |
|  |  | Reverse | CCCTCCAAAGCTGTGTGTATATCTC |  |  |  |

**References**

Alves E, Ovilo C, Rodriguez MC, Silio L. Mitochondrial DNA sequence variation and phylogenetic relationships among Iberian pigs and other domestic and wild pig populations. Anim Genet. 2003 Oct;34(5): 319–24. doi:10.1046/j.1365-2052.2003.01010.x. PMID: 14510666

Ramírez O, Ojeda A, Tomàs A, Gallardo D, Huang LS, Folch JM, et al. Integrating Y-chromosome, mitochondrial, and autosomal data to analyze the origin of pig breeds. Mol Biol Evol. 2009;26(9): 2061–72. doi:10.1093/molbev/msp118. PMID: 19535739
