# Supplementary material for: Contacts in the last 90,000 years over the Strait of Gibraltar evidenced by genetic analysis of wild boar (Sus scrofa)
Source: PLoS One. 2017 Jul 25;12(7):e0181929. doi: 10.1371/journal.pone.0181929 (PMC5526546; doi:10.1371/journal.pone.0181929)
Supplement: S3 Table — (A) The pairwise distances between clades are shown. (B) The pairwise distances between the haplotypes that belong to the European clade (E1). (DOCX) [file pone.0181929.s003.docx]

**S3 Table**. **The cytochrome b pairwise distances between sequences.**

(A) The pairwise distances between clades are shown. (B) The Pairwise distances between the haplotypes that belong to the European clade (E1).

1. ****

CAPTION: Estimates of Evolutionary Divergence over Sequence Pairs between Groups. The number of base substitutions per site from averaging over all sequence pairs between groups are shown in black. Standard error estimates are shown above the diagonal in blue. Analyses were conducted using the Tamura-Nei model [1]. The rate variation among sites was modeled with a gamma distribution (shape parameter = 0.5). The analysis involved 362 nucleotide sequences. Codon positions included were 1st+2nd+3rd+Noncoding. All positions containing gaps and missing data were eliminated. There were a total of 832 positions in the final dataset. Evolutionary analyses were conducted in MEGA6 [2].

1. ****

In Table B, CB9 and CB90 are the haplotypes found in North African wild boar. CB9 haplotype appears in samples from Africa, Europe, Near East and Asia. CB90 haplotype is exclusive to Morocco.

CAPTION: Estimates of Evolutionary Divergence between Sequences. The number of base substitutions per site from between sequences are shown in black. Standard error estimates are shown above the diagonal in blue. Analyses were conducted using the Tamura-Nei model [1]. The rate variation among sites was modeled with a gamma distribution (shape parameter = 0.5). The analysis involved 21 nucleotide sequences. Codon positions included were 1st+2nd+3rd+Noncoding. All positions containing gaps and missing data were eliminated. There were a total of 89 positions in the final dataset. Evolutionary analyses were conducted in MEGA6 [2].

**References**

1. Tamura K, Nei M. Estimation of the number of nucleotide substitutions in the control region of mitochondrial DNA in humans and chimpanzees. Mol Biol Evol 1993; 10:512-526.
2. Tamura K, Stecher G, Peterson D, Filipski A, Kumar S. MEGA6: Molecular Evolutionary Genetics Analysis version 6.0. Mol Biol Evol. 2013 Oct 16;30(12): 2725-9 doi: [10.1093/molbev/mst197](https://dx.doi.org/10.1093/molbev/mst197). PMID: 24132122; [PMC3840312](https://www.ncbi.nlm.nih.gov/pmc/articles/PMC3840312/)
