# Supplementary material for: Contacts in the last 90,000 years over the Strait of Gibraltar evidenced by genetic analysis of wild boar (Sus scrofa)
Source: PLoS One. 2017 Jul 25;12(7):e0181929. doi: 10.1371/journal.pone.0181929 (PMC5526546; doi:10.1371/journal.pone.0181929)
Supplement: S4 Table — (A) The pairwise distances between clades are shown. (B) The pairwise distances between the haplotypes that belong to the European clade (E1). (DOCX) [file pone.0181929.s004.docx]

**S4 Table**. **The control region pairwise distances between.**

(A) The pairwise distances between clades are shown. (B) The pairwise distances between the haplotypes that belong to the European clade (E1).

1.

CAPTION: Estimates of Evolutionary Divergence over Sequence Pairs between Groups. The number of base substitutions per site from averaging over all sequence pairs between groups are shown in black. Standard error estimates are shown above the diagonal in blue. Analyses were conducted using the Tamura 3-parameter model [1] with gamma distribution (shape parameter = 0.5). The differences in the composition bias among sequences were considered in evolutionary comparisons [2]. The analysis involved 1215 nucleotide sequences. All positions containing gaps and missing data were eliminated. There were a total of 348 positions in the final dataset. Evolutionary analyses were conducted in MEGA6 [3].

1.

CAPTION: Estimates of Evolutionary Divergence between Sequences. The number of base substitutions per site from between sequences are shown in black. Standard error estimates are shown above the diagonal in blue. Analyses were conducted using the Tamura 3- parameter model [1] with gamma distribution (shape parameter = 0.5). The analysis involved 60 nucleotide sequences. All positions containing gaps and missing data were eliminated. There were a total of 400 positions in the final dataset. Evolutionary analyses were conducted in MEGA6 [3].

**References**

1. Tamura K, Nei M. Estimation of the number of nucleotide substitutions in the control region of mitochondrial DNA in humans and chimpanzees. Mol Biol Evol. 1993 May;10(3): 512-526. PMID: 8336541
2. Tamura K, Stecher G, Peterson D, Filipski A, Kumar S. MEGA6: Molecular Evolutionary Genetics Analysis version 6.0. Mol Biol Evol. 2013 Oct 16;30(12): 2725-9 doi: [10.1093/molbev/mst197](https://dx.doi.org/10.1093/molbev/mst197). PMID: 24132122; [PMC3840312](https://www.ncbi.nlm.nih.gov/pmc/articles/PMC3840312/)
