# Supplementary material for: Contacts in the last 90,000 years over the Strait of Gibraltar evidenced by genetic analysis of wild boar (Sus scrofa)
Source: PLoS One. 2017 Jul 25;12(7):e0181929. doi: 10.1371/journal.pone.0181929 (PMC5526546; doi:10.1371/journal.pone.0181929)
Supplement: S5 Table — (DOCX) [file pone.0181929.s005.docx]

**S5 Table. Variable sites of the sequences obtained from Moroccan and Tunisian wild boars.**

| **Cytochrome-b sequences** | | | **Nucleotide positions**  (according to reference sequence) | | | | | | | |  | | | | |
| --- | --- | --- | --- | --- | --- | --- | --- | --- | --- | --- | --- | --- | --- | --- | --- |
| **Haplotypes** | |  | | 14,311 | 14,437 | 14,719 | 14,737 | 14,885 | 15,936 | 15,076 |  | | | | |
|  | | FJ237000 (Reference) | | G | T | G | C | A | C | G |  | | | | |
| CB90 | | Morocco A | | A | C | * | * | **·** | **·** | A |  |  |  |  |  |
| CB9 | | Morocco B | | **·** | **·** | * | * | **·** | **·** | **·** |  |  |  |  |  |
| CB9 | | WBMorocCytb1 and WBMorocCytb2 | | **·** | **·** | **·** | **·** | **·** | **·** | **·** |  |  |  |  |  |
| CB9 | | WBMorocCytb3 and WBMorocCytb4 | | **·** | **·** | A | **·** | * | **·** | **·** |  |  |  |  |  |
| CB9 | | WBMorocCytb5 | | **·** | **·** | **·** | - | G | G | **·** |  |  |  |  |  |
|  | | |  | | | | | | | |  | | | | |
| **Control region sequences** | | | **Nucleotide positions**  (according to reference sequence) | | | | | | | | | | | | |
| **Haplotypes** |  | | | 15,544 | 15,545 | 15,579 | 15,589 | 15,594 | 15,658 | 15,684 | 15,703 | 15,714 | 15,724 | 15,742 | 15,759 |
|  | FJ237000 (Reference) | | | G | T | A | C | A | A | G | C | A | A | C | C |
| CR1 | Morocco1, Morocco 4 and WBMorocCR 1 - 5 | | | · | **·** | **·** | **·** | **·** | **·** | **·** | **·** | **·** | **·** | **·** | **·** |
| CR140 | Morocco2 | | | **·** | **·** | **·** | **·** | **·** | **·** | **·** | T | G | **·** | **·** | **·** |
| CR141 | Morocco3 | | | **·** | **·** | **·** | **·** | **·** | **·** | A | **·** | **·** | G | T | **·** |
| CR181 | Tunisia 1 – 11 (except 6 and 10) | | | A | **·** | **·** | **·** | **·** | **·** | **·** | **·** | **·** | **·** | **·** | **·** |
| CR182 | Tunisia6 and Tunisia10 | | | **·** | C | - | T | G | G | **·** | T | **·** | **·** | **·** | T |

**Note:** We analysed cytochrome b between positions 14,223 and 15,269, and the control region between positions 15,452 and 15,858. We indicate sequence identities (dots (·)) and deletions (dashes (-)); asterisks (*) represent missing data). The cytochrome b and control region sequences from our study belong to the same wild boar when they have the same identification number. The nucleotide position numbers correspond to those in FJ237000 (complete European wild boar mtDNA).
