# Supplementary material for: Contacts in the last 90,000 years over the Strait of Gibraltar evidenced by genetic analysis of wild boar (Sus scrofa)
Source: PLoS One. 2017 Jul 25;12(7):e0181929. doi: 10.1371/journal.pone.0181929 (PMC5526546; doi:10.1371/journal.pone.0181929)
Supplement: S6 Table — (DOCX) [file pone.0181929.s006.docx]

**S6 Table. Estimated time of divergence calculated for each clade with strict clock**

|  |  | Estimated time of divergence | | | | |
| --- | --- | --- | --- | --- | --- | --- |
| Fragment | Model | *Phacochoerus africanus^a^* | Isolation between Asian clades (A) and the other clades (E1,E2,NE) | Isolation of the Italian clade (E2) | Isolation of the Near Eastern clade (NE) | Beginning of the isolation of the haplotypes found in North Africa*^C^* |
| Cytb | GTR + G | 7,000,000 (between  5,950,000 and 8,050,000) | 818,300 | 565,200 | 429,500*^b^* | 63,800 |
| Control Region | GTR+I+G | 6,751,824 (between  5,510,948 and 7,992,700) | 1,234,100 | 696,000^b^ | 857,100 | 116,600 |

*^a^*Divergence time between the genus *Sus* and *Phacochoerus africanus* (in years)*.*

*^b^*Isolation between this clade and the European clade (E1).

*^c^* This is the time of the isolation of the oldest haplotype found in North Africa to date.
